# A New Transgenic Tool to Study the Ret Signaling Pathway in the Enteric Nervous System

**DOI:** 10.3390/ijms232415667

**Published:** 2022-12-10

**Authors:** Ashoka Bandla, Ellie Melancon, Charlotte R. Taylor, Ann E. Davidson, Judith S. Eisen, Julia Ganz

**Affiliations:** 1 Department of Integrative Biology, Michigan State University, East Lansing, MI 48824, USA; 2 Institute of Neuroscience, University of Oregon, Eugene, OR 97403, USA; 3 Department of Biochemistry & Biophysics, University of California, San Francisco, San Francisco, CA 94143, USA

**Keywords:** Hirschsprung disease, neuronal development, enteric neuron, enteric progenitor cell, zebrafish, ENS neuropathies

## Abstract

The receptor tyrosine kinase Ret plays a critical role in regulating enteric nervous system (ENS) development. Ret is important for proliferation, migration, and survival of enteric progenitor cells (EPCs). Ret also promotes neuronal fate, but its role during neuronal differentiation and in the adult ENS is less well understood. Inactivating RET mutations are associated with ENS diseases, e.g., Hirschsprung Disease, in which distal bowel lacks ENS cells. Zebrafish is an established model system for studying ENS development and modeling human ENS diseases. One advantage of the zebrafish model system is that their embryos are transparent, allowing visualization of developmental phenotypes in live animals. However, we lack tools to monitor Ret expression in live zebrafish. Here, we developed a new BAC transgenic line that expresses GFP under the *ret* promoter. We find that EPCs and the majority of ENS neurons express *ret:GFP* during ENS development. In the adult ENS, GFP^+^ neurons are equally present in females and males. In homozygous mutants of *ret* and *sox10*—another important ENS developmental regulator gene—GFP^+^ ENS cells are absent. In summary, we characterize a *ret:GFP* transgenic line as a new tool to visualize and study the Ret signaling pathway from early development through adulthood.

## 1. Introduction

The receptor tyrosine kinase Ret forms a multicomponent receptor complex with a GDNF family receptor alpha (GFRalpha) subunit that binds several ligands including glial cell-line derived neurotrophic factor (GDNF). Ret is required for normal development of the enteric nervous system (ENS)—the intrinsic nervous system of the gut [1]. In humans, inactivating mutations in the proto-oncogene RET are the most common known cause of Hirschsprung disease—a congenital condition defined by loss of enteric neurons in the distal gut [2,3,4,5,6]. Functional loss of Ret in mice or zebrafish results in a lack of neurons in the ENS except for some neurons that remain in the anterior-most part of the gut in zebrafish [7,8,9,10,11]. In contrast, overactive RET signaling activity has been connected to multiple endocrine neoplasia (MEN) syndrome that includes ganglioneuromas of the gut [3,12].

At early stages of ENS development, enteric progenitor cells (EPCs) migrate to and along the gut [1]. During this stage, *ret* is expressed in migrating EPCs and Ret signaling promotes EPC proliferation, migration, and survival [8,13,14,15,16,17,18,19,20,21]. Ret continues to be expressed in neuronal EPCs and neurons into adulthood [15,20,22,23]. Ret signaling is critical for neuronal differentiation of bipotential EPCs, as shown by lineage analysis [19,23], and is also important for the survival of ENS neurons [14].

Zebrafish is an important research organism to study ENS development and function including the Ret signaling pathway [1,10,24,25]. Zebrafish are particularly well-suited for live imaging of changes in ENS development or function due to the embryo’s external development and transparency [1]. However, we do not have any transgenic tools that capture the whole extent of Ret signaling activity in live zebrafish in the ENS, as the existing *ret1:GFP* transgenic line is based on one *ret* enhancer, but does not contain all regulatory components of the *ret* gene that have been shown to drive expression in ENS cells [26]. A transgenic line that represents *ret* expression will enable us to monitor the effects of genetic or environmental perturbations on *ret* expressing ENS cells.

**Figure 1:**
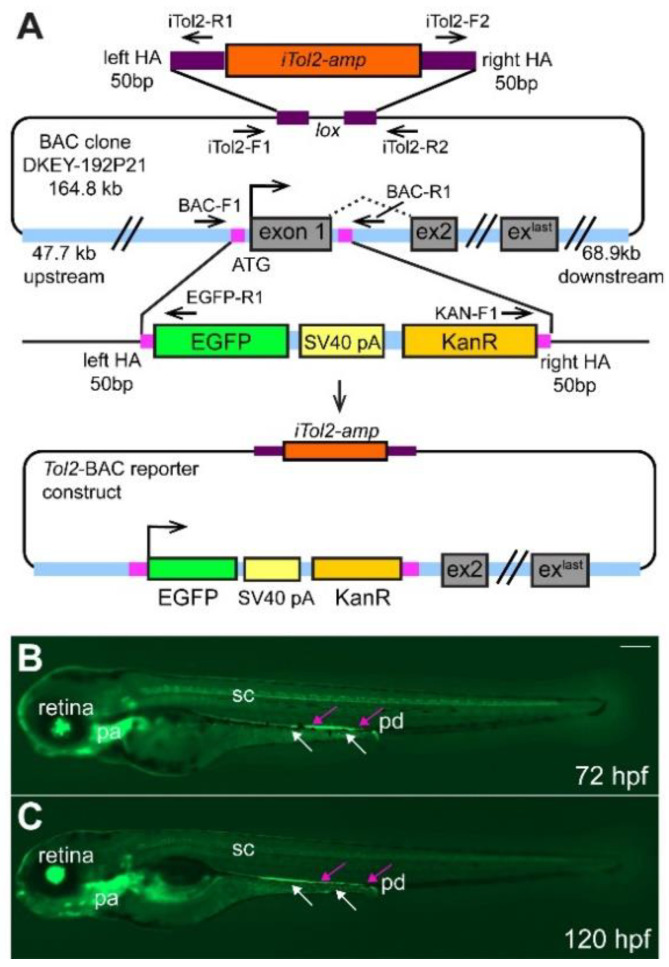
Overview of BAC-construct cloning strategy. (**A**) The start codon and exon 1 of *ret* were replaced by homologous recombination with the EGFP-SV40-pA-KanR construct using 50 bp-long homology arms (HA, magenta box) as indicated. iTol2 sites were included in the BAC backbone as shown using homologous recombination. The arrows indicate forward and reverse primer pairs to verify the correct generation of the tol2-BAC reporter construct. Integration of the BAC DNA does not result in overexpression of *ret* as the *ret* ATG is replaced by the EGFP cassette and the EGFP insert contains a strong transcription termination signal (SV40 pA, simian virus 40 poly A) [30]. Overview of GFP^+^ cells in the retina, pharyngeal arches (pa), spinal cord (sc), pronephric duct (pd, magenta arrows), and ENS cells (white arrows) at 72 (**B**) and 120 (**C**) hours post fertilization (hpf). (**B**,**C**): Whole-mount side-views of embryo/larva at the stage indicated. KanR kanamycin. Scale bar = 200 µm.

In this study, we developed and validated a new zebrafish BAC transgenic line *TgBAC(ret:EGFP)b1331* (referred to as *ret:GFP* from here forward) that expresses green fluorescent protein (GFP) under the *ret* promoter (Figure 1A). In addition to GFP^+^ ENS cells, other cells expressing GFP include cells in the developing pronephric ducts, enteroendocrine cells in the gut epithelium, neurons in the spinal cord and retina, and cells in the pharyngeal arches, in accordance with described *ret* expression in these cell types (Figure 1B,C, [8,15,27,28,29]). In this paper, we focus on *ret:GFP* expression in the ENS. We find GFP expression in migrating EPCs during early stages of ENS development. Later in ENS development, GFP is expressed in both ENS neurons and EPCs. We find GFP expression in two important neuronal subpopulations in the ENS, nitrergic and serotonergic neurons, confirming a broad role of Ret in regulating neuronal differentiation. For the first time, we identify GFP^+^ neurons in the adult zebrafish ENS, suggesting that *ret* function is also important in adult ENS neurogenesis. Analysis of the presence and distribution of GFP^+^ cells in zebrafish mutants of the ENS developmental regulators Ret or Sox10 reveals that GFP^+^ ENS cells are completely absent, suggesting that GFP^+^ ENS cells depend on the function of *ret* or *sox10* during development. In summary, our analysis puts forward the *ret:GFP* transgenic line as a new tool to visualize and study the Ret signaling pathway at all stages of ENS development and in adults.

## 2. Results

### 2.1. Generation of TgBAC(ret:EGFP)b1331 Transgenic Line

To generate the *TgBAC(ret:EGFP)b1331* transgenic line, the translational start codon of *ret* and exon 1 in the BAC clone DKEY-192P21 (GenBank accession number: BX005252.15) was replaced with an EGFP cassette (Figure 1A). For details, see also Material and Methods. Previously identified regulatory elements that drive expression in ENS cells [26] are included in the BAC clone, so we expect *TgBAC(ret:EGFP)b1331* transgenic line to reflect endogenous *ret* expression in the ENS. We compared mRNA expression of *ret* and *gfp* within the ENS at 48, 72, and 96 h post fertilization (hpf) and find comparable expression patterns (Figure S1), suggesting that *gfp* expression corresponds to *ret* expression in the ENS. However, EGFP protein has a half-life of approximately 24 h [31]. Therefore, GFP^+^ cells do not solely reflect *ret*-expressing cells but comprise cells that currently express *ret* as well as cells that have expressed *ret* but that still contain GFP protein. We have confirmed that the transgenic line consists of a single integration as it follows Mendelian transmission. In an outcross of a heterozygous *ret:GFP* carrier to wildtype, we found on average 50.7% GFP^+^ larvae per clutch (9 clutches, 744 embryos).

### 2.2. ret:GFP Is Expressed in Migrating Enteric Progenitor Cells and ENS Cells at Larval Stages

**Figure 2:**
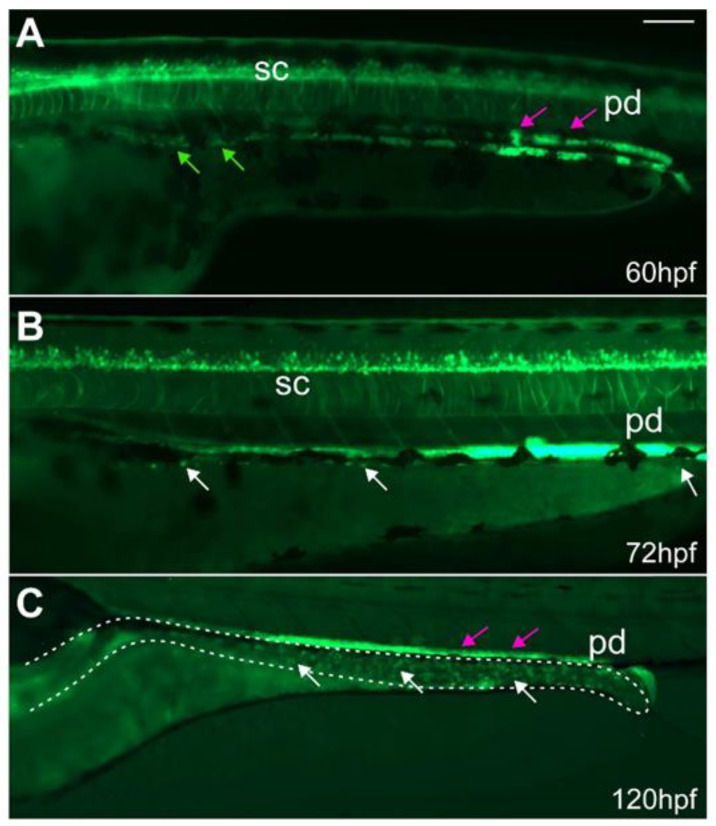
ret:GFP^+^ EPCs migrate along the developing gut and populate the gut. At (**A**) 60 hpf, GFP^+^ EPCs (green arrows) migrate along the developing gut. At (**B**) 72 and (**C**) 120 hpf, GFP^+^ ENS cells (white arrows) populate the gut (dashed line). Some of the GFP^+^ cells in the gut are enteroendocrine cells. The GFP^+^ pronephric ducts (pd, magenta arrows) directly overlay the ENS. (**A**–**C**): Whole-mount side-views of embryos/larvae at the stage indicated. Dashed line outlines the gut. Scale bar = 100 µm.

ENS development can be subdivided into two main stages: in the first, early stage of ENS development, neural-crest derived EPCs enter the gut at 32 hpf and migrate to and along the developing gut until they reach the posterior end around 66 hpf [1,32]. Starting at 54 hpf, ENS neurons start to differentiate from anterior to posterior. During this later stage of ENS development, EPCs proliferate and differentiate into different types of neurons or glial cells through larval stages until adulthood [1,32,33]. Early in ENS development, *ret* is expressed in migrating EPCs [8,15]. Thus, we first analyzed if migrating EPCs are GFP^+^ in *ret:GFP* transgenics. We found that at 60 hpf, GFP^+^ EPCs migrate along the gut in two parallel streams (Figure 2A). After EPC migration is completed, GFP^+^ cells were present within the ENS surrounding the gut at 72 and 120 hpf (Figure 2B,C). The ENS expression dynamics are in agreement with what has been previously described (Table 1, [8,15,28,34,35]).

### 2.3. The Majority of ret:GFP Cells in the Gut Are ENS Neurons

**Figure 3:**
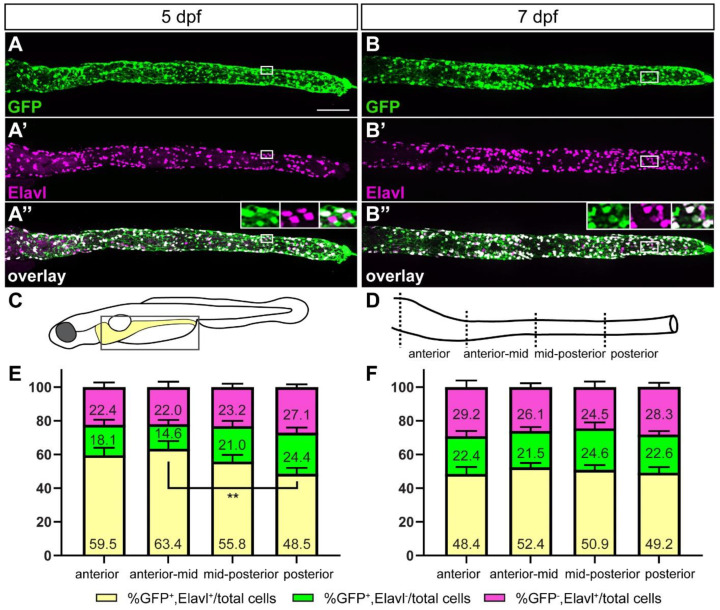
The majority of GFP^+^ cells are ENS neurons at later stages of ENS development. At 5 (**A**–**A”**) and 7 (**B**–**B”**) days post fertilization (dpf) GFP^+^ ENS neurons (white) are found along the whole length of the gut. A smaller fraction are GFP^+^ non-neuronal cells (green) or GFP^−^ ENS neurons (magenta). Insets in (**A”**,**B”**) show the close-up of the boxed area, GFP, Elavl, and overlay from left to right. Schematic of boxed area of larval schematic (**C**) indicates the four gut sub-regions analyzed (**D**). Quantification of GFP and Elavl colocalization at 5 dpf (**E**) and 7 dpf (**F**) in four gut regions shown in (**D**). Bar graph shows % GFP^+^/Elavl^+^ out of total number of cells quantified [GFP^+^/Elavl^+^, GFP^+^/Elavl^−^, and GFP^−^/Elavl^+^], (yellow), % GFP^+^/Elavl^−^ out of total cells (green), and % GFP^−^/Elavl^+^ out of total cells (magenta). Using 2-way ANOVA, we did not find significant differences between the cell populations of the different gut regions except where indicated (** *p* ≤ 0.01). Error bars show ±standard error of the mean (5 dpf: 2 experiments, 19 larvae; 7 dpf: 2 experiments, 15 larvae). (**A**–**A”**,**B**–**B”**): maximum projections of dissected guts at stage indicated. Scale bar = 100 µm.

During later stages of zebrafish ENS development, *ret* has been shown to be expressed in enteric neurons [15]. Thus, we hypothesized that *ret:GFP* expressing cells are ENS neurons. To test this hypothesis, we performed double immunostaining for GFP and the pan-neuronal marker Elavl at 5 dpf (Figure 3A–A”,C–E). In 5-day-old larvae, we see three distinct cell populations: GFP^+^/Elavl^+^, GFP^+^/Elavl^−^, and GFP^−^/Elavl^+^ (Figure 3A–A”). More specifically, we found that 73.2 ± 3.2% of GFP colocalizes with Elavl, defining the neuronal cell population. The 26.8% ± 3.2% cells that express GFP but are Elavl negative comprise the non-neuronal cell population. GFP^+^ enteroendocrine cells were differentiated from GFP^+^ ENS neurons by their location in the gut epithelium and their characteristic teardrop shape and thereby not included in the non-neuronal population.

To determine if there are regional differences in the distribution of GFP^+^ ENS neurons, we subdivided the gut into four regions: (1) anterior, (2) anterior-mid, (3) mid-posterior, and (4) posterior part (Figure 3D). The percentage of GFP^+^ ENS neurons was not significantly different in the anterior, anterior-mid, and mid-posterior parts of the gut (Figure 3E). In the posterior region, however, we saw significantly fewer GFP^+^ ENS neurons compared to the anterior-mid region (Figure 3E). We also found a non-significant trend towards more GFP^+^ but Elavl^−^ ENS cells posteriorly (Figure 3E). In addition, we noticed that a portion of ENS neurons was GFP^−^ in all four gut regions, but this feature was more pronounced in the posterior part of the gut (Figure 3E).

To determine if the distribution of GFP^+^ ENS neurons along the gut changes over time, we quantified colocalization of GFP and Elavl at 7 dpf and found the same three cell populations identified at 5 dpf (Figure 3B–B”,C,D,F). At 7 dpf, GFP^+^ ENS neurons are evenly distributed along the length of the gut with no significant differences between the different gut regions. We also found a population of GFP^−^ ENS neurons that showed equal distribution along the length of the gut (Figure 3F). At this stage, the size of the non-neuronal cell population of GFP^+^/Elavl^−^ cells was comparable to what has been observed previously [33].

### 2.4. ret:GFP Is Expressed in Two Prominent Neuronal Subpopulations

**Figure 4:**
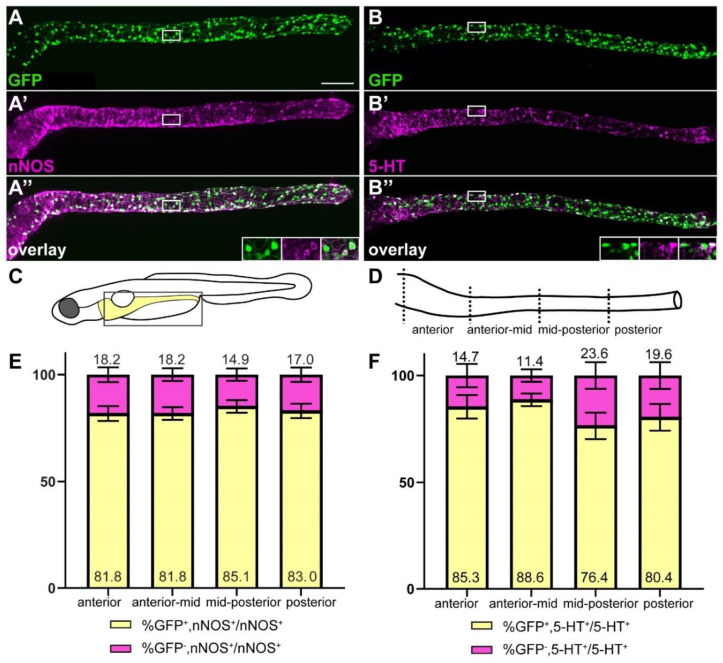
The majority of nitrergic and serotonergic neurons are GFP^+^. *ret:GFP* is expressed in the majority of nitrergic (**A**–**A”**) and serotonergic (**B**–**B”**) neurons (white). Insets in (**A”**,**B”**) show close-up of the boxed area, GFP, nNOS or 5-HT, and overlay from left to right. Schematic of boxed area of larval schematic (**C**) indicates the four gut sub-regions analyzed (**D**). (**E**) Quantification of GFP and nNOS colocalization in four gut regions as shown in (**D**). Bar graph shows % GFP^+^/nNOS^+^ out of total nNOS^+^ cells (yellow), and % GFP^−^/nNOS^+^ out of total nNOS^+^ cells (magenta). Error bars show ±standard error of the mean (2 experiments, 23 larvae). (**F**) Quantification of GFP and 5-HT colocalization in four gut regions shown in D. Bar graph shows % GFP^+^/5-HT^+^ out of total 5-HT^+^ cells (yellow), and % GFP^−^/5-HT^+^ out of total 5-HT^+^ cells (magenta). Using 2-way ANOVA, we did not find significant differences between the cell population of the different gut regions. Error bars show ±standard error of the mean (2 experiments, 22 larvae). (**A**–**A”**,**B**–**B”**): maximum projections of dissected guts. Scale bar = 100 µm.

Since we found that the majority of GFP^+^ cells are ENS neurons, we wanted to determine if two specific neuronal subtypes—nNOS^+^ nitrergic and 5-HT^+^ serotonergic neurons—are included in the GFP-positive cell population. To address this question, we performed co-staining of GFP and 5-HT or nNOS at 5 dpf. We found that most nitrergic and serotonergic neurons are GFP^+^ (Figure 4). The proportion of GFP^+^ nitrergic and serotonergic neurons is evenly distributed along the gut with no statistically significant differences between the different regions of the gut (Figure 4C–F). A small portion of nitrergic and serotonergic neurons are GFP^−^ (Figure 4E,F), indicating that those neuronal subtypes are present in both the GFP^+^ and the GFP^−^ neuronal populations.

### 2.5. GFP^+^ Cells Include Proliferating EPCs

**Figure 5:**
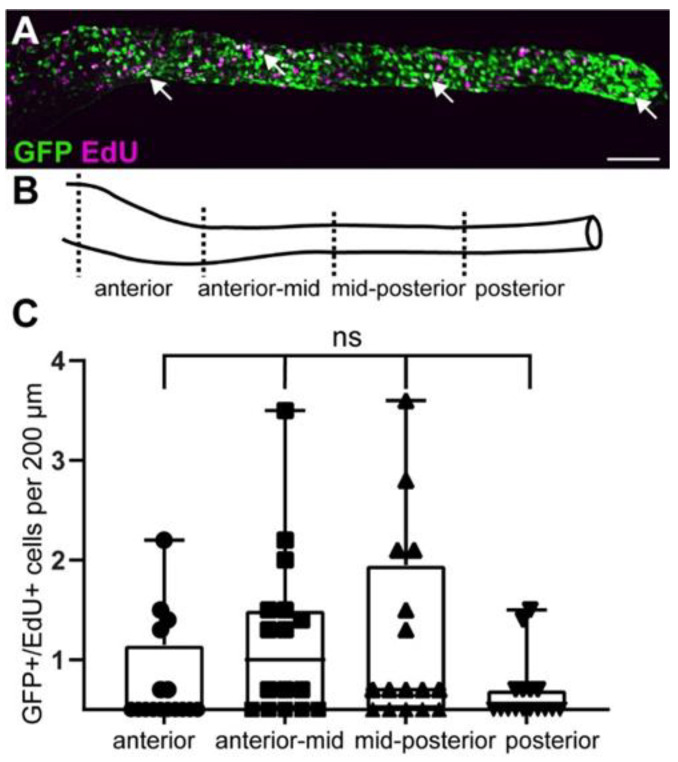
GFP^+^ cells include proliferating enteric progenitor cells at larval stages. (**A**) At 5 dpf, GFP colocalizes with EdU (white arrowheads). (**B**) Gut schematic indicates the four gut sub-regions analyzed. (**C**) Quantification of GFP and EdU colocalization in four gut regions shown in (**B**). Using one-way Anova, we did not find significant differences between the different gut regions. Box plot shows GFP^+^/EdU^+^ cells (2 experiments, 16 larvae). (**A**): maximum projection of dissected gut. Scale bar = 100 µm. ns not significant.

Next, we wanted to determine if GFP^+^ cells comprise proliferating EPCs at larval stages. Based on the expression of *ret* in EPCs during earlier stages of ENS development, we hypothesized that a subset of GFP^+^ cells also proliferate at larval stages. To test this hypothesis, we performed double immunostaining with GFP and EdU after a short 4 h EdU pulse. EdU is a thymidine analog and is incorporated in DNA during S-phase and thus labels proliferating cells [36]. We then analyzed GFP and EdU colocalization in the anterior, anterior-mid, mid-posterior, and posterior sections of the gut (Figure 5). At 5 dpf, GFP colocalizes with EdU across the length of the gut, which suggests that GFP^+^ cells include proliferating EPCs. We found no significant difference in the distribution of GFP^+^/EdU^+^ cells between the different gut regions.

### 2.6. Adult ENS Neurons Express ret:GFP

**Figure 6:**
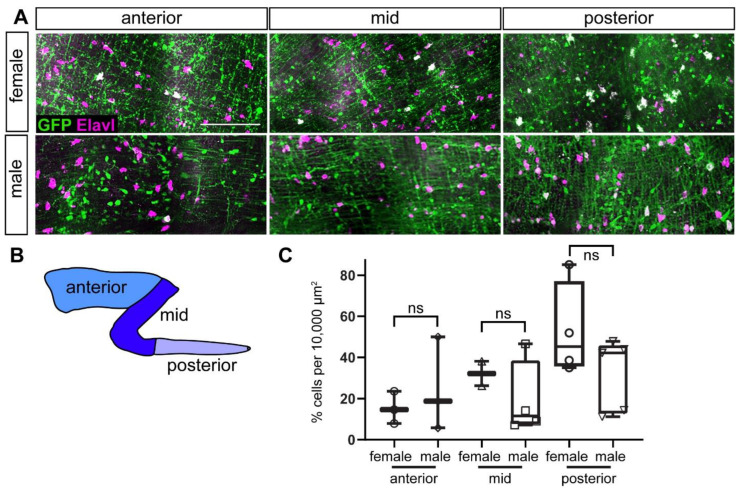
ENS neurons are GFP^+^ in adults. (**A**) GFP^+^ ENS neurons (white) at different anterior-posterior levels along the gut in male and female adult zebrafish. (**B**) Schematic of gut subdivisions. (**C**) Quantification of colocalization of GFP and Elavl in males and females in the three gut regions. Using *t*-tests, we did not find significant differences between males and females in the different gut regions. Box plot shows % GFP^+^/Elavl^+^ out of total Elavl cells per 10,000 µm^2^ (≥2 guts per condition). (**A**): maximum projections of dissected guts. ns not significant. Scale bar = 100 µm.

To test whether *ret:GFP* is expressed in adult ENS neurons, we performed double immunostaining with GFP and Elavl in adult guts. We quantified colocalization of GFP and Elavl in the anterior, mid, and posterior sections of the gut (Figure 6). A smaller percentage of ENS neurons are GFP^+^ in adults than in larvae, ranging from an average 12.3% to 47.5% depending on the gut region (Figure 6C). When we compared numbers between females and males, we found no significant difference in GFP^+^ ENS neurons in all three gut regions.

### 2.7. ret:GFP Positive ENS Cells Are Absent in ret and sox10 Mutants

**Figure 7:**
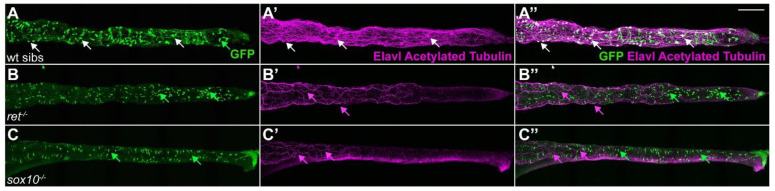
*ret* and *sox10* mutants lack GFP^+^ ENS cells. (**A**–**A”**) In 6 dpf wildtype siblings (wt sibs), GFP^+^ cells colocalize with the pan-neuronal markers Elavl and acetylated Tubulin (AT, white arrows) in ENS neurons; Elavl^−^/AT^−^/GFP^+^ cells with a characteristic teardrop shape are enteroendocrine cells (green arrow). In 6 dpf *ret* (**B**–**B”**) or *sox10* (**C**–**C”**) homozygous mutants, GFP^+^ ENS neurons are absent. AT^+^ processes are the vagal innervation to the gut (magenta arrows). Elavl^−^/AT^−^/GFP^+^ enteroendocrine cells are still present (green arrows). We analyzed ≥30 larvae per genotype. (**A**–**C**), maximum projections of dissected guts. Scale bar = 100 µm.

To understand whether our *ret:GFP* line faithfully represents ENS cells, we tested if GFP^+^ cells are present in two zebrafish lines that carry null mutations in the known ENS developmental regulator genes, *ret* and *sox10*. Homozygous mutants for the *ret ^hu2846^* allele only have a few remaining ENS neurons in the anterior region of the gut. Homozygous mutants for the *sox10^t3^* allele completely lack enteric neurons [9,10,37]. To analyze the presence and distribution of GFP^+^ cells in homozygous *ret* or *sox10* mutants, we crossed the *TgBAC(ret:EGFP)b1331* line to heterozygous carriers of the *ret* or *sox10* mutations. We then incrossed *ret:GFP^+^* heterozygous carriers of the *ret* or *sox10* mutations and analyzed their offspring. To detect ENS neurons, ENS axonal tracts, and the extrinsic vagal nerve tracts, we performed double immunostaining with GFP, Elavl, and acetylated Tubulin, which labels neuronal projections (Figure 7A, [38]). In wildtype, we found the expected GFP^+^/Elavl^+^/acetylated Tubulin^+^ ENS neurons (Figure 7A–A”, white arrows). In *ret* and *sox10* homozygous mutants, we found that Elavl^+^/acetylated Tubulin^+^ ENS neurons were consistently absent. The remaining AT^+^ processes in *ret* and *sox10* homozygous mutants comprise the vagal innervation to the gut (Figure 7B–B”,C–C”, magenta arrows). Analysis of the distribution of GFP^+^ cells in the gut showed that *ret* mutants did not have any GFP^+^ ENS cells but still had GFP^+^ enteroendocrine cells. Likewise, *sox10* homozygous mutants lacked GFP^+^ ENS cells but still have GFP^+^ enteroendocrine cells (Figure 7B–B”,C–C”, green arrows). These observations suggest that our transgenic line is representative of known *ret* expression in the ENS because, in the absence of *ret* or *sox10* gene function, GFP^+^ ENS cells are absent.

## 3. Discussion

The Ret signaling pathway plays a critical role during ENS development. Appropriate levels of Ret activity are crucial for EPC proliferation, migration, and survival [8,13,14,15,16,17,18,19,20,21]. In the ENS, migration, and proliferation are closely connected; decreased proliferation of EPCs leads to decreased EPC migration and vice versa [39,40,41]. As Ret signaling impacts both EPC migration and proliferation, inactivating mutations of Ret have profound effects on EPC gut colonization. The importance of Ret for proper ENS development is reflected in the prevalence of mutations in RET in Hirschsprung disease patients. Imbalance of Ret signaling activity is also implicated in MEN syndrome [3,12]. Despite the importance of Ret signaling in ENS development, we know little about its role in neuronal differentiation both during development and in adulthood. Additionally, we lack tools to monitor Ret expressing cells in zebrafish, an important model for studying factors that control ENS development and function, and what goes awry in situations that model human ENS diseases.

This study presents and validates a *ret:GFP* transgenic zebrafish line that represents a new tool for understanding the role of Ret during ENS development. Studying the distribution of GFP^+^ cells and the cell types that express *ret:GFP* in the ENS, we have five main findings: (1) the majority of GFP^+^ cells in the ENS are neurons at larval stages, (2) GFP^+^ ENS neurons are present in the adult zebrafish ENS, albeit at a lower percentage than at larval stages, (3) a small percentage of ENS neurons are GFP^−^ at larval and adult stages (4) GFP^+^ cells include EPCs, and (5) GFP^+^ ENS cells are absent from the guts of *ret* and *sox10* mutants. The new *ret:GFP* transgenic line described in this study will be an important tool to study Ret signaling in zebrafish at all stages of ENS development and adulthood in a live animal.

### 3.1. Most Enteric Neurons Are GFP Positive

In mice and human, single-cell RNA-sequencing analysis found that *Ret* is expressed in most ENS neurons in juveniles and adults [22,42,43,44]. Accordingly, a *ret:EGFP* transgenic mouse line showed EGFP expression in differentiating neurons [21]. Ret signaling activity is essential for neuronal fate in the ENS. In mice, EPCs that differentiate into ENS neurons sustain *Ret* expression, whereas EPCs on the path toward glial differentiation downregulate *Ret* [23]. In zebrafish, *ret* is expressed in a subpopulation of ENS neurons during ENS neurogenesis [15,35]. Our analysis shows that the majority of *ret:GFP* expressing ENS cells are neurons at larval stages, which agrees with the previously observed expression patterns of *ret* and underscores that Ret is important for neuronal development in the zebrafish ENS. Fewer ENS neurons express *ret* in the posterior region of the gut at 84 hpf [15]. Consistent with this observation, we find fewer GFP^+^ ENS neurons in the posterior gut at 5 dpf. At 7 dpf, the percentage of GFP^+^ ENS neurons is equal in the different parts of the gut. Since ENS neurons differentiate from anterior to posterior along the gut [1,32], our quantification suggests that a differentiation gradient might still be detectable at 5 days, but has evened out at 7 dpf.

### 3.2. Adult ENS Neurons Are GFP Positive

In mammals, Ret continues to be expressed in a population of ENS neurons in the adult and contributes to synapse formation and neuronal plasticity by regulating neurite outgrowth [42,43,45,46,47,48]. Recently, it has been shown that ENS neurogenesis continues into adulthood in zebrafish [33], but which signals regulate neuronal development at that stage remains unknown. We find GFP^+^ ENS neurons in the adult, which suggests that Ret may play a role in regulating ENS neurogenesis into adulthood. Female and male guts had a comparable distribution of GFP^+^ ENS neurons, suggesting that there is no sex difference connected to the expression of *ret* in the adult. This is consistent with reports that found no difference in Ret expression in ENS neurons in adult female or male mice [43].

### 3.3. A Small Proportion of ENS Neurons Is GFP-Negative at Larval and Adult Stages

From larval stages and in adulthood, we find a small population of GFP^−^ ENS neurons along the gut length. This is consistent with what has been observed in adult human and mouse ENS, which also contain Ret^+^ and Ret^−^ populations of ENS neurons in the adult [22,43,44,49]. Interestingly, the percentage of GFP^−^ ENS neurons increases in the adult gut compared to larval stages, suggesting that in the adult Ret-independent ENS neurogenesis is more prevalent than in larval zebrafish.

### 3.4. Enteric Progenitor Cells Are GFP-Positive at Larval Stages

The presence of proliferative cells in the zebrafish ENS and their detailed molecular profile, particularly at later stages of ENS development, remains unresolved. During their migration toward and along the gut, EPCs express different genes including *phox2bb*, *sox10*, and *ret* [8,15,50]. The molecular profile of EPCs at larval stages, however, has not been determined, particularly the question of whether EPCs continue to express *ret*. In this study, we find GFP^+^ cells that are proliferating at 5 dpf, suggesting that *ret-*expressing EPCs continue to be present at later stages of ENS development. This is contrary to previous reports that suggest that there is no resident proliferating cell population in the zebrafish ENS at postembryonic stages [51]. Our analysis shows that a non-neuronal cell population comprises 26.8% of GFP^+^ cells at 5 dpf and 31.5% at 7 dpf. Undifferentiated or glial EPCs are part of the GFP^+^ non-neuronal cell population. Neuronal EPCs that have committed to a neuronal fate start to express Elavl at early stages of neuronal differentiation, thus some of the GFP^+^ EPCs may also be Elavl^+^. A subset of the GFP^+^/Elavl^−^ cells could also be enteric glial cells that were just born from GFP^+^ EPCs. As GFP persists in cellular offspring for a certain time [31], ENS glia could still be GFP^+^, even though they have downregulated *ret* expression [23,42]. Recently, cells with glial characteristics have been identified as progenitor cells in the zebrafish gut. These cells express *her4.3:GFP* [33]. It will be interesting to determine if the GFP^+^ EPCs are within the same progenitor cell population as the *her4.3:GFP* positive cells or if different subpopulations of EPCs are present in the zebrafish larval gut.

## 4. Materials and Methods

### 4.1. Zebrafish Husbandry and Strains

All experiments were carried out in accordance with animal welfare laws, guidelines, and policies and were approved by Michigan State University Institutional Animal Care and Use Committee and the University of Oregon Institutional Animal Care and Use Committee. *TgBAC(ret:EGFP)b1331* zebrafish and heterozygous carriers of *ret^hu2846^* and *sox10^t3^* [9,37] were maintained in a laboratory breeding colony according to established protocols [52]. *ret^hu2846^* (ZFIN ID: ZDB-ALT-070315-12) and *sox10^t3^* (ZFIN ID: ZDB-GENO-130429-1) animals were of the AB background. We identified homozygous *ret^hu2846^* mutants by morphological criteria as previously described [10,53] and homozygous *sox10* mutants by lack of pigmentation [37]. Adult zebrafish were bred naturally in system water and fertilized eggs were transferred to 100 mm Petri dishes containing ~25 mL of embryo medium. Embryos were allowed to develop at 28.5 °C and staged by hours post fertilization according to morphological criteria [54,55].

### 4.2. Generation of Transgenic Line

To generate the *TgBAC(ret:EGFP)b1331* transgenic line, the translational start codon of *ret* in the BAC clone DKEY-192P21 (GenBank accession number: BX005252.15) was replaced with an EGFP-SV40-pA-KAN cassette essentially as previously described [30,56]. For recombination, each homologous arm flanking the EGFP-SV40-pA-KAN cassette was 50 bp long. The 5′ homologous arm was 3 bp upstream of the start codon of the *ret* gene and the 3′ homologous arm 541 bp downstream of the start codon. Itol2 sites were included in the BAC backbone using the same technology [30,57,58]. The final BAC construct was coinjected with Tol2 transposase mRNA into one-cell stage zebrafish embryos as described previously with minor modifications [30,56]. BAC DNA was co-injected at a concentration of 50 ng/µL and Tol2 transposase mRNA at a concentration of 35 ng/µL. Approximately 1–2 nL (with 2% phenol red) was injected directly into the cytoplasm of 1-cell stage zebrafish embryos. To confirm the correct integration of the BAC DNA construct, we performed PCR across the integration sites as indicated in the diagram (Figure 1A).

### 4.3. Tissue Preparation

*TgBAC(ret:EGFP)b1331* larvae were fixed in 4% paraformaldehyde (PFA) in 1X sweet buffer (8% sucrose; 0.2 M CaCl_2_; 0.2 M PO_4_ buffer) at pH 7.3 for 2 h at room temperature. Adult fish (10–12 months old) with an average body length of ~3 cm for both sexes and gut length of on average 2.1 cm for the females and 1.7 cm for males, were sacrificed and then dissected carefully to collect the entire intestine. The intestine was first placed in 1X PBS solution and then gently placed on a filter paper to remove fat tissue. The intestine was quickly transferred back to the 1X PBS and sectioned into 3 parts—anterior, mid, and posterior—as described previously [59]. Sectioned intestines were then fixed in 4% PFA in 1X sweet buffer for 2 h at room temperature. They were then rinsed in 0.5% PBS-Triton^TM^ X-100 followed by immunostaining.

### 4.4. Immunostaining

Double immunostaining of larvae and adult guts for 5-HT (1:10,000, Immunostar, Hudson, WI, USA, catalog number 20080), Acetylated Tubulin (1:2000, Sigma-Aldrich, St. Louis, MO, USA, catalog number T6793), Elavl (1:1000, Thermo Fisher Scientific, Eugene, OR, USA, catalog number A-21271), GFP (1:1000, Thermo Fisher Scientific, Eugene, OR, USA, catalog number A-11120), GFP (1:1000, Invitrogen/Thermo Fisher Scientific, Eugene, OR, USA, catalog number A11122), and nNOS (1:1000, GeneTex, Irvine, CA, USA, catalog number GTX133407) was performed as previously described [38] with minor modifications. Briefly, embryos or adult tissues were rinsed five times with double distilled water for 1 h each and then incubated in a blocking solution for an hour at room temperature. The blocking solution was prepared with 0.5% PBS-Triton^TM^ X-100, 1% DMSO, 2% bovine serum albumin (BSA, Sigma-Aldrich, catalog number A3059), and 5% normal growth serum (NGS, ThermoFisher Scientific, catalog number PCN5000). Embryos or adult tissues were then incubated for 16–18 h in primary antibodies in blocking solution. After 3 washes of a minimum of 60 min each in 0.3% PBS Triton^TM^ X-100, the embryos or adult tissues were incubated for 16–18 h in secondary antibodies in blocking solution. Finally, embryos or adult tissues were washed 3 times for a minimum of 60 min each and stored in the refrigerator in 0.3% PBS Triton^TM^ X-100.

### 4.5. In Situ Hybridization

Antisense-RNA probe templates were obtained by NotI linearization of *ret* plasmid [8,27], and PCR amplification of GFP from genomic DNA isolated from finclips of *TgBAC(ret:EGFP)b1331* carriers with primers containing T7 promoter sequence (T7: 5′-TAATACGACTCACTATAGGG-3′). Primer sequences were EGFP_ISH_F1: 5′-CAAGGGCGAGGAGCTGTT-3′, EGFP_ISH_R1: 5′-TAATACGACTCACTATAGGG CTCGTCCATGCCGAGAGT-3′. Template DNA was purified with the Clean and Concentrate kit (Zymo Research Corporation, Irvine, CA, USA, catalog number D4014) and T7 RNA polymerase (Promega Corporation, Madison, WI, USA, catalog number P2075) was utilized for the transcription reaction to generate DIG-labeled antisense RNA probe. The whole-mount RNA in situ hybridization was performed essentially as previously described [60] with minor modifications. Briefly, 2, 3, and 4 dpf zebrafish embryos/larvae were fixed in 4% PFA in 1X sweet buffer for 2 h at room temperature for 2 and 3 dpf, and overnight at 4 °C for 4 dpf larvae. Samples were then rinsed in 0.5% PBS-Triton X-100 followed by a depigmentation procedure. For depigmentation, embryos were treated with 3% H_2_O_2_ and 0.5% KOH for varying times based on the developmental stage (2 dpf: 30 min, 3 dpf: 50 min, and 4 dpf: 65 min). Then, embryos were washed in 0.1% PBS Tween 20 (PBSTw) for 5 min to remove the H_2_O_2_. Embryos were then progressively dehydrated by washing for 5 min in each of the following concentrations of methanol—25%, 50%, 75% in 0.1% PBSTw—followed by 5 min wash in 100% methanol. Samples were then stored at −20 °C in 100% methanol. Embryos were rehydrated in a decreasing series of methanol 75%, 50%, 25% in 1X 0.1% PBSTw. Permeabilization was achieved by incubating with Proteinase K (Roche Diagnostics, Indianapolis, IN, USA, catalog number 1092766) in 0.1% PBSTw at room temperature (RT) under the following conditions: 2–3 dpf embryos were incubated in 10 µg/mL proteinase K for 30 min and 4 dpf larvae in 20 µg/mL proteinase K for 45 min. This was followed by 3 quick rinses with 1X 0.1% PBSTw and 20 min 4% PFA fixation at RT. Pre-hybridization occurred for 2–4 h at 68 °C in hybridization solution (50% formamide, 5X SSC, 50 ug/mL Heparin, 500 µg/mL tRNA, 0.1% Tween 20, 9.2 mM Citric Acid). Hybridization with 50 ng DIG-labeled probe (antisense *gfp* and antisense *ret*) was carried out overnight in 68 °C water bath as previously described [60]. Stringency washes at 68 °C were carried out with 1× hybridization solution, 2× hybridization solution (without tRNA and heparin), 2 × 10 min 2X SSCTw (2X SSC/0.1% Tween 20, 2 × 30 min 0.2X SSCTw, 2 × 2 h 2X SSCTw as previously described [61]. Washes at RT included 1 × 5 min 66% 2X SSCTw in PBSTw and 1 × 5 min 33% 2X SSCTw in PBSTw and 1X PBSTw. Samples were incubated in block (5% sheep serum (Sigma-Aldrich, St. Louis, MO, USA, catalog number S3772), 2 mg/mL BSA (Sigma-Aldrich, catalog number A3059) in 0.1% PBSTw) at RT for 1–4 h followed by incubation with anti-DIG-AP primary antibody (1:5000; Roche Diagnostics, Indianapolis, IN, USA, catalog number 11093274910) in block overnight at 4 °C on a nutator. After 8 × 10 min washes in 0.1% PBSTw, embryos were washed 3 × 5 min in alkaline Tris buffer (100 mM Tris-Cl, 50 mM MgCl_2_, 100 mM NaCl, 0.1% Tween 20 in DEPC treated water). Embryos were then placed in staining solution containing 250 µg/mL Nitro Blue Tetrazolium (NBT, Sigma-Aldrich, St. Louis, MO, USA, catalog number N6639) and 175 µg/mL 5-bromo 4-chloro 3-indolyl phosphate (BCIP, Sigma-Aldrich, St. Louis, MO, USA, catalog number B-8503) in alkaline Tris buffer until a strong signal was achieved.

### 4.6. EdU Staining

Zebrafish larvae were incubated with 500 µM EdU in embryo medium for 4 h at 28.5 °C and then fixed in 4% PFA in PBS for 3 h at room temperature followed by EdU detection and immunostaining. EdU incorporation was detected using the Click-iT^TM^ Plus EdU Cell Proliferation Kit for Imaging, Alexa Fluor^TM^ 555 dye (ThermoFisher Scientific, catalog number C10638). Larvae were treated with 250 µL of EdU reaction cocktail for 1 h at room temperature in the dark.

### 4.7. Image Acquisition

Confocal images were acquired on a 3i spinning disk confocal microscope using a 20× objective and Slidebook6 software. Low magnification images were acquired using a Zeiss AxioZoom fluorescent dissecting microscope and LAS software. Images were processed and analyzed using FIJI software [v2.3.0/1.53f, [62]], Adobe Photoshop 2020 (Version 21.0.2, Adobe Systems, Inc., San Jose, CA, USA), and Adobe Illustrator 2020 (Version 24.0.2, Adobe Systems, Inc, San Jose, CA, USA).

### 4.8. Image Analysis

Following immunohistochemistry, intestines were dissected and mounted onto a glass slide in 0.3% PBS Triton^TM^ X-100 and covered with a coverslip. For cell quantification, z-stacks were generated from confocal stacks in Slidebook6. These z-stacks were used for manual cell counts and colocalization analysis. GFP^+^ Enteroendocrine cells were differentiated from GFP^+^ ENS neurons by their location in the gut epithelium and their characteristic teardrop shape. For the larval gut, counts were performed by measuring the length of the gut and dividing the intestine into four equal sections labeled as anterior, anterior-mid, mid-posterior, and posterior. To allow for comparison between guts of different lengths, cell counts were normalized to 200 µm. For adult gut tissue, the different sections of the tissue were randomly imaged. To quantify, two to five images per gut section were marked with four randomly placed boxes of 100 × 100 µm^2^. These boxes were used for manual cell counts and colocalization analysis.

### 4.9. Statistical Analysis

To determine significant differences, we performed an unpaired *t*-test. For multiple comparisons, we performed one-way-ANOVA or two-way-ANOVA followed by Tukey’s post-hoc test using GraphPad Prism 9.1.0, San Diego, CA, USA.

## Figures and Tables

**Table 1:** Overview of *ret* expression patterns during ENS development.

Stage of ENS Development		hpf	ENS Cell Type	Method of Detection	Reference
EPC migration ^a^		36	EPCs ^c^	RNA in situ hybridization	[8,15]
	neuronal differentiation ^b^	54	EPCs ^c^ + neurons ^d^	RNA in situ hybridization	[15]
		60	EPCs ^c^ + neurons ^d^	RNA in situ hybridization	[15]
		68–70	neurons ^e^	scRNA-seq	[35]
		72	neurons ^e^	RNA in situ hybridization	[28]
		84	neurons ^d^	RNA in situ hybridization	[15]
		168	neurons ^e^	bulk RNA-seq experiment	[34]

^a^ EPCs enter the developing gut at 32 hpf and reach the posterior end at ~66 hpf, the timeline of EPC migration is marked in green [32]; ^b^ Neuronal differentiation (timeline is marked in orange) starts at 54 hpf and occurs from anterior to posterior [32]; ^c^ quantification shows that subset of EPCs expresses *ret*; ^d^ quantification shows that subset of neurons expresses *ret*; ^e^ extent of *ret* expression not quantified in cell type. scRNA-seq single-cell RNA-sequencing; RNA-seq RNA-sequencing.

## Supplementary Materials

The following supporting information can be downloaded at: https://www.mdpi.com/article/10.3390/ijms232415667/s1.
