# Supplementary material for: A New Transgenic Tool to Study the Ret Signaling Pathway in the Enteric Nervous System
Source: Int J Mol Sci. 2022 Dec 10;23(24):15667. doi: 10.3390/ijms232415667 (PMC9779438; doi:10.3390/ijms232415667)
Supplement: Supplementary file 1 [file ijms-23-15667-s001.zip › ijms-1789707-supplementary.pdf]

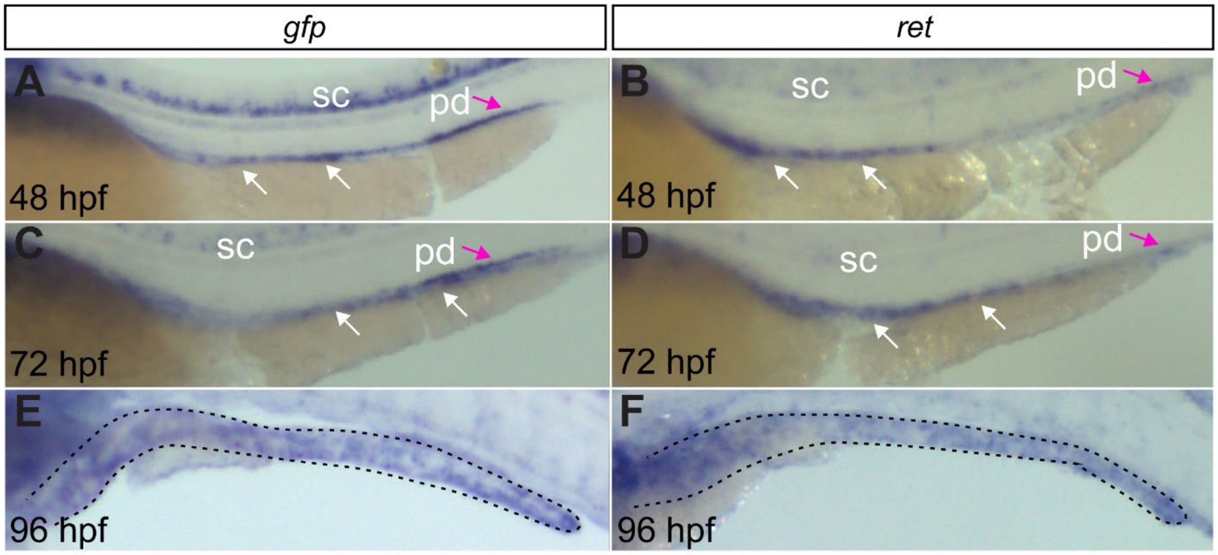

Figure S1. *gfp* and *ret* expression (purple) is comparable in ENS cells (white arrows) at 48 hpf (A, B), 72 (C, D), and 96 (E, F) hours post fertilization (hpf). At 48 and 72 hpf, the pronephric ducts (pd, magenta arrows) are close to the ENS. sc spinal cord. A-F: Whole-mount side views at the stage indicated.
